# Surgical Management of Acute Rockwood Grade III Acromioclavicular Joint Dislocations: A Systematic Review

**DOI:** 10.7759/cureus.28657

**Published:** 2022-09-01

**Authors:** Isaac Okereke, Elsenosy Abdelfatah

**Affiliations:** 1 Trauma and Orthopaedics, The Royal London Hospital, London, GBR; 2 Trauma and Orthopaedics, University Hospitals Dorset, Poole, GBR

**Keywords:** acj management, acromioclavicular joint, acj dislocation, type iii acj injuries, rockwood grade iii

## Abstract

Injuries of the acromioclavicular joint (ACJ) occur frequently in young and active people. The best management of acute grade III injuries has been a source of controversy and extensive debate. When surgery is indicated, there is still no gold standard surgical technique for treating acute grade III ACJ injuries.

The methodology of this review was a comprehensive search of PubMed, Medline, Cochrane, and EMBASE databases using various combinations of the keywords “Rockwood,” “type III,” “grade III,” “treatment,” “surgery,” “acromioclavicular joint,” and “dislocation,” since the inception of the databases to December 2020. Surgical techniques were divided into two groups. In group 1 were ACJ fixation techniques using hardware such as the hook plate, Kirschner wires, and wire cerclage; group 2 included coracoclavicular (CC) ligament fixation/reconstruction techniques using double buttons, TightRope®, suture anchors, Endobuttons, the Infinity-Lock^TM^ Button System, etc. Fourteen studies were selected for the final review following the Preferred Reporting Items for Systematic Reviews and Meta-Analyses (PRISMA) guidelines.

This review showed better outcome scores in group 2. Overall, complication rates were higher in group 1 compared to group 2. The results of this review show that CC fixation, using suspensory or loop devices, of Rockwood grade III injuries, has better outcomes and fewer complications than fixation of the ACJ with hardware.

## Introduction and background

Injuries of the acromioclavicular joint (ACJ) are common in young and active people and account for about 9% of all shoulder girdle injuries [1]. The literature shows the incidence rate to be considerably higher in young athletes involved in throwing and contact sports such as rugby, football, and wrestling (up to 40% of shoulder injuries) and in manual laborers. ACJ injuries occur about five times more frequently in men compared to women [2].

The acromioclavicular joint is an amphiarthrodial joint formed at the articulation of the lateral clavicle with the acromion. Essentially attaching the shoulder girdle to the axial skeleton and capable of very complex, previously misunderstood movements [3], the ACJ relies on static and dynamic stabilizers. The acromioclavicular (AC) ligament comprising anterior, posterior, superior, and inferior ligaments is the primary static stabilizer of the acromioclavicular joint. Further stability is provided by the coracoclavicular (CC) ligaments (conoid and trapezoid ligaments) [4]. Several classification systems have been developed to aid the diagnosis and appropriate management of ACJ dislocations. The original classification by Tossy, Mead, and Sigmond [5] was adopted and revised by Rockwood, Williams, and Young, and three further subgroups were added [6] to the Tossy type III group. The Rockwood classification of ACJ injuries (Table 1) is based on the degree of soft tissue injuries and the direction of the displacement of the clavicle from the acromion established on an AP radiograph of the shoulder [7] ideally taken at an angle of 10°-15° with both arms hanging down and displaying both shoulders in the same film. Stress radiographs with patients holding a 5-8 kg weight in the affected arm can also be done to differentiate a Rockwood grade II from a grade III injury [8].

**Table 1:** Rockwood classification of acromioclavicular dislocations AC, acromioclavicular; CC, coracoclavicular; CCD, coracoclavicular distance

Classification of injury	AC ligaments	CC ligaments	Deltopectoral fascia	CCD
Type I	Sprained	Intact	Intact	Normal
Type II	Disrupted	Sprained	Intact	<25% of the normal side
Type III	Disrupted	Disrupted	Disrupted	25%-100% of the normal side
Type IV	Disrupted	Disrupted	Disrupted	Posterior dislocation
Type V	Disrupted	Disrupted	Disrupted	>100% of normal side
Type VI	Disrupted	Disrupted	Disrupted	Decreased

The goal of the treatment of an acute ACJ dislocation is to restore the dislocated joint’s normal anatomy and alignment and permit a return to full power without any limitations on the range of movement during activity [9]. Rockwood grade I and II injuries have historically been managed non-operatively with a sling initially for comfort and then active rehabilitation. In contrast, types IV, V, and VI are generally managed operatively [10,11]. A grade III injury is characterized by a complete tear of the acromioclavicular (AC) and coracoclavicular (CC) ligaments, detachment of the deltotrapezial fascia from the distal clavicle, and an increase of between 25% and 100% of the coracoclavicular distance (CCD) when compared to the normal contralateral shoulder on an AP radiograph [12].

The best surgical management of grade III injuries is a source of ongoing controversy and debate [11,13,14], and there is still a lack of evidence to support current treatment options [15]. On whether to manage operatively or not, Smith et al. determined through a meta-analysis of relevant studies that there was statistically no significant difference in clinical or radiological outcomes between operative and non-operative management in patients with a type III injury [14]. Furthermore, in a systematic review of eight studies that compared operative with non-operative management of grade III injuries, Korsten et al. found better objective and subjective shoulder function outcomes in the operative group. However, while the operative group had higher complication rates and radiographic abnormalities, the non-operative group had an averagely shorter rehabilitation time with poorer cosmetic outcomes [13]. A Cochrane review reported a need for good‐quality randomized trials of currently used surgical interventions versus conservative treatment for well‐defined injuries, finding insufficient evidence between the two treatments in pain at one year, treatment failure usually resulting in secondary surgery, or patient satisfaction with the cosmetic result [16]. When the decision is made to manage operatively, there is no gold standard surgical technique for the treatment of acute grade III injuries.

This systematic review aims to compare the outcomes of the different surgical techniques employed, when indicated, in the treatment of grade III ACJ injuries.

## Review

Methods and materials

Ethical approval and patient consent were not required in this review as it is based on previously published studies.

Search Strategy and Study Selection

**Figure 1:**
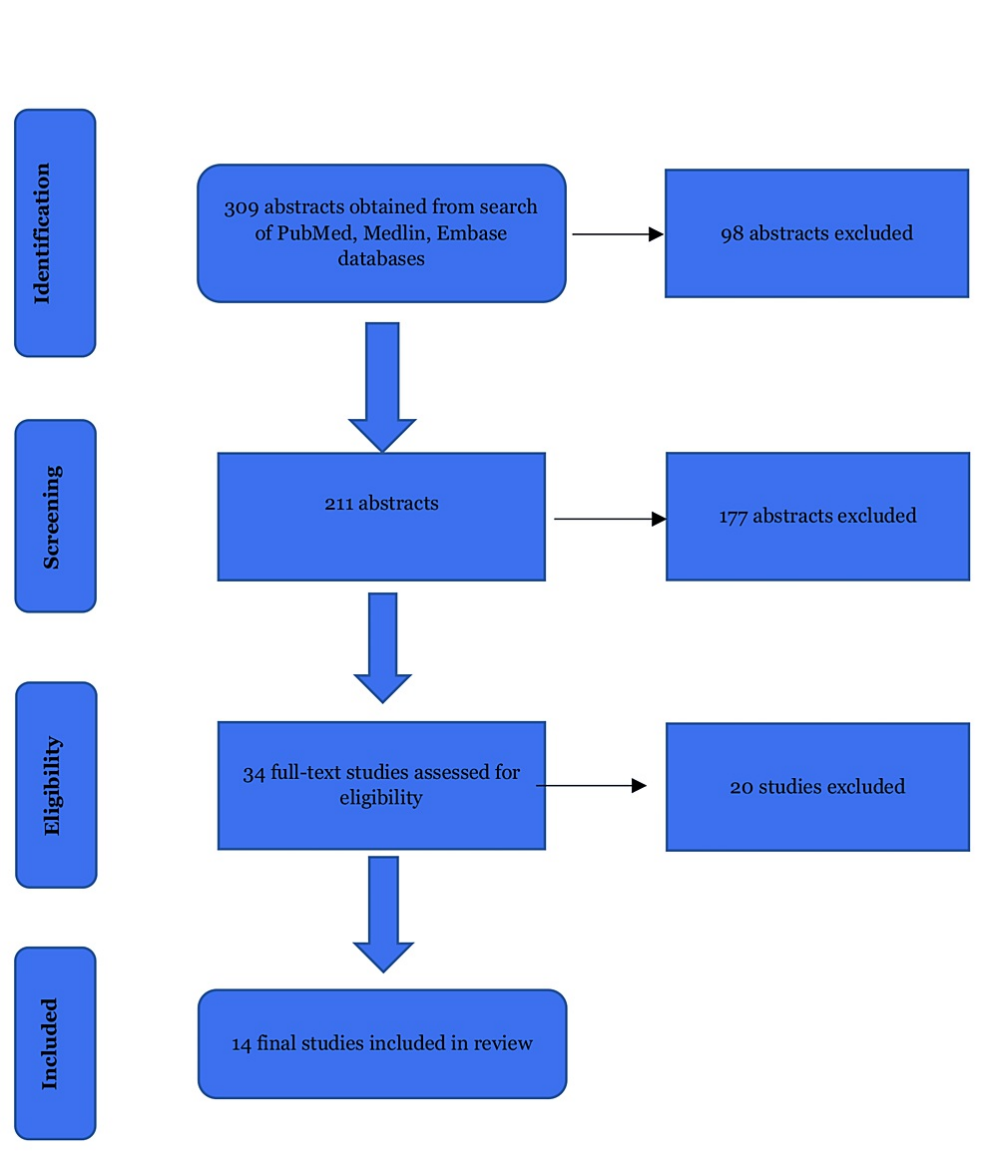
PRISMA study selection flow diagram PRISMA, Preferred Reporting Items for Systematic Reviews and Meta-Analyses

PubMed, Medline, Cochrane, and EMBASE databases were searched since inception following the Preferred Reporting Items for Systematic Reviews and Meta-Analyses (PRISMA) guidelines (Figure 1) [17] by two independent reviewers (IO and EA) using various combinations of the keywords “Rockwood,” “type III,” “grade III,” “treatment,” “surgery,” “acromioclavicular joint,” and “dislocation.” The inclusion criteria were full-text articles in the English language published in a peer-reviewed journal that reported a Rockwood grade III injury of the acromioclavicular joint and clearly described surgical management, follow-up duration, outcomes, and complications. Selected publications had cross-referencing done to obtain relevant articles that met the predetermined criteria of inclusion. We excluded narrative reviews, case reports, animal and cadaver studies, technical and instructional notes, letters to editors, and articles with no full text available or not published in English.

Data Extraction

Two investigators (IO and EA) performed the data extraction using a standardized Excel file (Microsoft Corp., Redmond, WA). A third reviewer (MA) confirmed the data extracted by the two independent reviewers and reviewed the final study. The following data were extracted from the included studies: first author’s name, year of publication, study design, surgical technique used, the sample size in each group, patient characteristics/demographics, duration of follow‐up, outcome measures, and complications. Surgical procedures were divided broadly into two groups: group 1 included techniques that involve fixation of the ACJ using hardware, e.g., K-wires, wire cerclage, and hook plates, and group 2 comprised procedures requiring repair of the CC ligament by either rigid or non-rigid fixation devices and anatomical or non-anatomical CC ligament reconstruction/augmentation by autogenous or synthetic grafts. Any disagreements between the investigators were resolved by discussion and consensus.

Outcome Measures and Statistics

The outcome measures of interest were the Constant-Murley (CM) score [18], Taft score [19], Oxford Shoulder Score (OSS) [20,21], disabilities of the arm, shoulder, and hand (DASH) scores [22], pain on the visual analog scale (VAS) [23], coracoclavicular distance (CCD) [24], University of California Los Angeles Shoulder (UCLA) score [25], revisions, and other complications. Statistical analysis was done using SPSS version 27.0 (IBM Corp., Armonk, NY, USA). A p-value of <0.05 was considered significant.

Results

The search query resulted in 306 abstracts, with three more included from cross-referencing selected studies making a total of 309 abstracts. 98 abstracts were excluded due to duplicate records leaving 211 abstracts. After screening titles/abstracts for satisfying inclusion/exclusion criteria, 177 abstracts were further excluded, leaving 34 unique studies for full-text review. Finally, 14 studies that reported outcomes after the surgical management of acute Rockwood grade III acromioclavicular dislocations were included in this systematic review (Figure 1) [12,19,26-37].

Demographics and Study Characteristics

There were two level I studies, two level II studies, and 10 level III studies included in this review. A combined total of 590 patients with an acute grade III ACJ dislocation divided equally between the two groups were included in the final 14 studies included in this study. The mean age at the time of surgery and follow-up duration was 39.84 years and 79.9 months, respectively, in group 1 and 36.4 years and 37 months in group 2 (Table 2). The gender distribution of the patient cohorts was not reported in five studies. The surgical techniques reported in group 1 were hook plate fixation, ACJ fixation with a wire cerclage, the modified Phemister technique, and fixation using Steinmann pins. The surgical techniques described in group 2 included rigid fixation of the CC ligament using a Bosworth screw, non-rigid fixation with sutures in the Ladermann procedure, utilizing the TightRope™ system, Endobuttons, and reinforcement with a semitendinosus tendon graft (Table 3). A level I study by Ye et al. compared the use of hook plates and an autologous semitendinosus tendon graft in patients with a grade III ACJ injury directly [34]. Darabos et al., in a prospective randomized double-blind clinical trial, compared minimally invasive CC fixation with TightRope® implant to Bosworth screws in 68 patients with grade III AC dislocations [26]. The use of hook plates without CC ligament reconstruction in the surgical treatment of grade III injuries was reported by Kumar and Sharma in a prospective study of military personnel [30].

**Table 2:** Patient characteristics of (groups 1 and 2)

Study variable	Group 1	Group 2
Number of studies	9	9
Number of patients	295	295
Mean time to surgery (days)	12.6	14.4
Mean age (years)	39.84	36.4
Mean time to follow-up (months)	79.9	37
Complications reported (%, number)	75% (222)	20.6% (61)

**Table 3:** Study and patient characteristics HP, hook plate; ST, semitendinosus; WD, Weaver-Dunn; TR, TightRope®; MP, modified Phemister; DB, double button; CCDT, common closed‑loop double‑endobutton technique; MCDT, modified closed‑loop double‑endobutton technique

Author	Study design (level of evidence)	Number	Surgical technique (number of patients)	Mean age (years)	Time of follow-up	Time to surgery
Darabos et al. [26]	Prospective, randomized, double-blind clinical trial (I)	68	TightRope® (34), Bosworth screw (34)	TightRope® group, 37.25 ± 11.75; Bosworth screw group, 41.18 ± 14.1	6 months	2 weeks
De Carli et al. [27]	Retrospective study (III)	30	TightRope® system	28.7	3.5 years (range, 2-8 years)	7 days
Łazarski et al. [28]	Prospective study (II)	27	Wire cerclage (12), Ladermann method (11), hook plate (4)	39.26 ± 12.84	22.83 months	2 weeks
Calvo et al. [29]	Retrospective study (III)	32	MP, 21	39.6 (18-68)	122.8 months (range, 12-228 months)	<3 weeks
Kumar et al. [30]	Prospective study (II)	33	Hook plate	34.24 (21-55)	23.5 months (range, 20-26 months)	<48 hours
Leidel et al. [31]	Retrospective study (III)	70	K-wires	37 ± 11	Group A, 1-2 years; group B, 3-5 years; group C, 6-10 years	<3 weeks
Lizaur et al. [12]	Retrospective study (III)	38	K-wires	57 (41-71)	21 years	<72 hours
Taft et al. [19]	Retrospective study (III)	52	Steinmann pins (26), Bosworth screws (26)	11-79	10.8 years	<3 weeks
Topal et al. [32]	Retrospective study (III)	20	Suture anchor (9), DB (11)	Suture anchor, 39 (24-56); DB, 37 (22-50)	Suture anchor group, 12.7 months (range, 12-16 months); DB, 13.8 months (range, 12-21 months)	
Wang et al. [33]	Retrospective study (III)	60	TightRope® system (30), Endobutton system (30)	TightRope® group, 39.37 ± 15.31; Endobutton system, 42.20 ± 13.49	2 years	<3 weeks
Ye et al. [34]	Prospective, randomized, double-blind clinical trial (I)	46	Hook plate group (23), autologous ST graft (23)	Hook plate group, 33.4 ± 3.3; semitendinosus graft group, 34.3 ± 3.3	12 months	<4 days
Muñoz García et al. [35]	Retrospective study (III)	21	MP, 21	34 (18-59)	57 months (range, 27-96 months)	<3 weeks
Cetinkaya et al. [36]	Therapeutic study (III)	32	Bosworth screws (16), K-wires (16)	Bosworth screws, 38 (24-52); K-wires, 53.3 (38-64)	96 months, 93 months	<3 weeks
Zhang et al. [37]	Retrospective study (III)	61	Modified DB (20), DB (21), hook plate (20)	CCDT, 30.25 ± 7.41; MCDT, 29.90 ± 6.9; HP, 30.55 ± 8.04	>1 year	<7 days

Outcomes

The Constant-Murley (CM) score was the most frequently reported outcome measure recorded in nine studies (Table 4 and Table 5). The average CM score for group 1 was 87.2 and that for group 2 was 91.9.

Łazarski et al. reported statistically significant improved CM scores in patients randomized to techniques in group 2 compared to the use of hook plates in group 1 (p = 0.04) [28]. Ye et al. and Zhang et al. also compared the use of hook plates (group 1) to a semitendinosus graft plus Endobuttons and the modified/common closed-loop double-button technique (group 2), respectively, both reporting generally better functional outcomes in patients who had the group 2 procedures [34,37]. Taft et al. reported outcomes using a 4-point scale of subjective, objective, and X-ray findings postoperatively. The average reported score for patients in group 1 was 9.0 compared to 9.7 for group 2 [19]. The OSS, reported in only two studies, was an average of 42.16 in group 1 and 44.43 in group 2. An average UCLA score of 31.55 was reported in two studies in group 1 and 34 studies in group 2.

Łazarski et al. reported Patient-Reported Outcomes Measurement Information System (PROMIS) scores. Patients who had fixation using the Ladermann technique (group 2) had average PROMIS scores of 86.45 (66-90), while those who had fixation using a hook plate and wire cerclage (group2) had average PROMIS scores of 80.25 (61-89) and 89.5 (87-90), respectively. A comparison of the preoperative and postoperative CCD was reported in five studies, with one study reporting only the preoperative CCD. A detailed comparison of the outcomes in the two groups is shown in Table 5.

**Table 4:** Outcome scores of patients treated with fixation of the ACJ with hardware HP, hook plate; MP, modified Phemister; PROMIS, Patient-Reported Outcomes Measurement Information System; ASES, American Elbow and Shoulder Surgeons; N/R, not recorded; SPADI, Shoulder Pain and Disability Index; DASH, disabilities of the arm, shoulder, and hand; UCLA, University of California Los Angeles Shoulder; CCD, coracoclavicular distance; SD, standard deviation; VAS, visual analog scale; ACJ, acromioclavicular joint

Author	Surgical technique	Number	Outcome scores						
			Constant score	DASH score	Oxford Shoulder Score	Imatani scores	UCLA score	Radiologic outcome CCD preoperative/postoperative (mm)	Others
Ye et al. [34]	HP	23	80.4	N/R	N/R	N/R	N/R	N/R	N/R
Łazarski et al. [28]	HP	4	78.97 (range, 69.5-96.5; SD, 12.10)	N/R	38.75 (range, 21-46; SD, 11.89)	N/R	N/R	N/R	PROMIS, 80.25 (range, 61-89; SD, 12.99)
	Wire cerclage	12	95.22 (range, 68-100; SD, 10.33)	N/R	45.58 (range, 39-48; SD, 2.64)	N/R	N/R	N/R	PROMIS, 89.5 (range, 87-90; SD, 1)
Calvo et al. [29]	MP	32	N/R	N/R	N/R	93.7 ± 9.9	N/R	N/R	N/R
Kumar et al. [30]	Hook plate	33	91.8 (95% CI, 88.5-93.05)	N/R	N/R	N/R	32.3 (95% CI, 31.9-32.6)	N/R	N/R
Leidel et al. [31]	K-wires, group A (1-2 years) (follow-up)	70	88 ± 13	N/R	N/R	N/R	N/R	N/R	ASES score, 27 ± 6; SPADI scores, 5 ± 15
	Group B (3-5 years) (follow-up)		89 ± 10	N/R	N/R	N/R	N/R	N/R	29 ± 2, 2 ± 6
	Group C (6-10 years) (follow-up)		86 ± 7	N/R	N/R	N/R	N/R	N/R	29 ± 2, 1 ± 3
Lizaur et al. [12]	Temporary K-wires + suture of the deltoid and trapezius over the clavicle	38	N/R	89.1 (range, 36-100)	N/R	91.9 (range, 64-100)	30.8 (range, 12-35)	17.6 ± 5.0/<1.5 mm in 76%	N/R
Taft et al. [19]	Steinmann pins	26	N/R	N/R	N/R	N/R	N/R	N/R	4-point scale, 9.0
Zhang et al. [37]	HP	20	N/R	N/R	N/R	N/R	N/R	N/R/16.83 ± 0.75	ASES, 44.25 ± 2.55
Cetinkaya et al. [36]	MP	16	89 (45-100)	N/R	N/R	N/R	N/R	N/R	VAS, 3 (2-4)
Muñoz García et al. [35]	MP	21	N/R	89	N/R	N/R	N/R	17.10/12.10	N/R

**Table 5:** Outcome scores of patients treated with rigid/non-rigid CC fixation/reconstruction techniques DB, double button; MDB, modified double button; TR, TightRope®; SA, suture anchor; EB, Endobutton; IL, Infinity-Lock Button System; SAC, Specific Acromioclavicular Joint score; N/R, not recorded; DASH, disabilities of the arm, shoulder, and hand; UCLA, University of California Los Angeles Shoulder; ASES, American Elbow and Shoulder Surgeons; ACJI, acromioclavicular joint instability; PROMIS, Patient-Reported Outcomes Measurement Information System; CCDT, common closed‑loop double‑endobutton technique; MCDT, modified closed‑loop double‑endobutton technique

Author	Surgical technique	Number	Outcome scores						
			Constant score	DASH score	Oxford Shoulder Score	Imatani scores	UCLA score	Radiologic outcome	Others
Cetinkaya et al. [36]	Bosworth screw	16	86 (70-100)	N/R	N/R	N/R	N/R	N/R	N/R
Darabos et al. [26]	Bosworth screw	34	87.42	9.9	43.17	N/R	N/R	25.44/19.22	N/R
	TR system	34	92.22	6.46	44.59	N/R	N/R	26.94/15.74	N/R
De Carli et al. [27]	TR system	30	98.2 ± 2.8	N/R	N/R	N/R	34 ± 0.9		ASES score, 100; ACJI score, 87.9 ± 2.2
Łazarski et al. [28]	Ladermann method	11	95.22 (range, 68-100; SD, 10.33)	N/R	45.54 (range, 35-48; SD, 4.18)	N/R	N/R	N/R	PROMIS score, 86.45 (range, 66-90; SD, 8.14)
Taft et al. [19]	Bosworth screw	26	N/R	N/R	N/R	N/R	N/R	N/R	4-point scale, 9.7
Topal et al. [32]	SA	9	89.6 (50-98)	6.65 (0-38.3)	N/R	N/R	N/R	19 (14-30)/15.7 (9.8-18.8)	N/R
	DB	11	93.6 (90-98)	2.48 (0-4.2)	N/R	N/R	N/R	19 (12-30)/16 (9.6-23.1)	N/R
Ye et al. [34]	ST graft and EB	23	90.3 ± 5.4	N/R	N/R	N/R	N/R	N/R	N/R
Wang et al. [33]	EB system	30	93.27 ± 1.59	N/R	N/R	N/R	N/R	23.57 ± 2.69/11.47 ± 1.19	N/R
	TR system	30	93.70 ± 1.78	N/R	N/R	N/R	N/R	23.50 ± 2.08/11.40 ± 1.13	N/R
Zhang et al. [37]	CCDT	21	N/R	N/R	N/R	N/R	N/R	N/R /16.0 ± 0.77	N/R
	MCDT	20	N/R	N/R	N/R	N/R	N/R	N/R /16.77 ± 0.91	N/R

Complications

Postoperative loss of reduction/redislocation, pain, infection, CC ligament ossification, and osteoarthritis were the most frequently reported surgery complications. Table 6 shows a detailed comparison of surgical complications between groups 1 and 2. The mean infection rate in group 1 was 3.38% (n = 10) and that in group 2 was 1.6% (n = 5). Of the patients in group 1, 16% (n = 48) reported a loss of reduction at follow-up compared to 6% (n = 17) in group 2. Of the patients who had a fixation with K-wires or Steinmann pins (n = 134), 20% (n = 28) reported pin migration on follow-up.

**Table 6:** Postoperative complications OA, osteoarthritis; N/R, not recorded; MP, modified Phemister; ACJ, acromioclavicular joint

Group 1							
Author	Complications						
	Surgical technique	Number of patients	Failure/loss of reduction	Infection	CC ligament ossification	OA	Others
Cetinkaya et al. [36]	MP	16	6.5% (n = 2)	12% (n = 2)	12% (n = 2)	18% (n = 3)	
Calvo et al. [29]	MP	32	50% (n = 16)	3% (n = 1)	59% (n = 19)	80% (n = 26)	ACJ deformity, 9% (n = 3); osteolysis of the lateral clavicle, 43% (n = 14)
Leidel et al. [31]	K-wire fixation	70	11.5% (n = 8)	N/R	N/R	N/R	K-wire migration, 4% (n = 3)
Lizaur et al. [12]	K-wire fixation	38	13% (n = 5)	N/R	N/R	N/R	OA, 28% (n = 11)
Taft et al. [19]	Steinmann pins	26	15% (n = 4)	7.6% (n = 2)	N/R	N/R	Pin migration, 32.6% (n = 11); fixation devise breakage, 23% (n = 6); arthritis, 35% (n = 9)
Ye et al. [34]	Hook plate	23	8.7% (n = 2)	N/R	N/R	N/R	Acromial osteolysis, 8.7% (n = 2)
Muñoz García et al. [35]	MP	21	33% (n = 7)	23% (n = 5)	N/R	N/R	Pin migration, 65.4% (n = 17); hypertrophic scar, 46.2% (n = 12)
Group 2							
Darabos et al. [26]	Bosworth screw	34	11.76% (n = 4)	N/R	N/R	N/R	Screw breakage, 11.76% (n = 4)
	TightRope®	34	5.88% (n = 2)	N/R	N/R	N/R	N/R
De Carli et al. [27]	TightRope®	30	N/R	3% (n = 1)	70% (n = 21)	N/R	Dislocation of TightRope®, 3% (n = 1)
Taft et al. [19]	Bosworth screw	26	40% (n = 11)	15% (n = 4)	N/R	15% (n = 4)	Miscellaneous, 7% (n = 2)
Cetinkaya et al. [36]	Bosworth screw	16	12% (n = 2)	0%	18% (n = 3)	12% (n = 2)	N/R

Discussion

Several factors determine the indication for primary surgical fixation of acute grade III injuries, and they include pre-injury function, post-injury functional deficit, the presence of functional pain, age, sex, hand dominance, and the patient’s preference for operative intervention. The optimal surgical treatment of grade III ACJ dislocations is still controversial, and a topic of debate among surgeons, as the current evidence, is inconclusive. Although different surgical techniques have been described in the literature for the management of ACJ joint dislocations, controversies regarding the timing of surgery, open versus arthroscopic surgery, and the choice of surgical procedure are still prevalent, more so for grade III injuries. The purpose of this study was to review the literature to determine the best surgical treatment for acute grade III acromioclavicular injuries.

The surgical repair of an ACJ dislocation can be grouped into techniques that allow either fixation of the ACJ and achievement of fixation between the coracoid and the clavicle or a reconstruction/augmentation of the CC ligament.

The goal of ACJ fixation is to stabilize the ACJ and reduce the CCD to allow the healing of ligaments disrupted by injury. ACJ fixation was historically achieved using Kirschner wires or Steinmann pins as described by Taft et al. and Leidel et al. [19,31]. As shown in this review, the severe complications of pin migration, potential damage to close neurovascular structures, and osteoarthritis have made the use of these devices unacceptable in current practice [38,39]. The hook plate is now the most used device to achieve fixation at the ACJ. Proponents of this technique argue that with a hook plate inserted at the ACJ, there is a non-rigid fixation at the CC joint that permits for the implant to be left in longer to allow for sufficient healing of the AC and CC ligaments and the commencement of early active abduction of the shoulder [40]. In spite of its widespread acceptance, the loss of reduction of CCD is a concern associated with implant removal after hook plate fixation for ACJ dislocation. Other complications of using a hook plate are the need for repeat surgery, persistent shoulder pain, incomplete shoulder function, acromial osteolysis, and acromioclavicular subluxation [30]. It is also now well established that the AC ligaments provide biomechanical stability against horizontally directed forces, while the CC ligaments are the main resistors to vertical forces [41]. The drawback of procedures that reconstruct or fix only the AC ligamento-capsular complex is that they do not provide sufficient resistance to vertical displacement that is usually provided by the CC ligaments. Furthermore, these techniques have the potential of damaging the intra-articular structures of the joint [42].

Fixation between the clavicle and coracoid can be achieved by either rigid or non-rigid devices. Excellent outcomes have been reported in the use of Bosworth screws to achieve rigid fixation in grade III injuries [19,26,36]. In the Bosworth technique, a half-threaded cancellous screw with a pulling effect that passes through both cortices of the coracoid and clavicle is inserted in an open procedure and routinely removed at six to eight weeks to prevent screw breakage or migration. Cetinkaya et al. reported good outcomes by also repairing the CC ligament with Ethicon before the insertion of a Bosworth screw as recommended by Rockwood [36,43].

Non-rigid fixations of the CC ligament have the benefit of eradicating the need for a second surgery for implant removal and include the use of polydioxanone sulfate (PDS), suture anchors, TightRope®, and Ethibond sutures. The TightRope® was first described as a technique for the treatment of AC dislocation in 2007 [26]. Darabos et al. reported better outcomes with the use of TightRope™ compared to Bosworth screws in the treatment of acute ACJ dislocations [26].

With recent advancements in surgical instrumentation and arthroscopic techniques, there has been an increase in the use of arthroscopically assisted or all-arthroscopic reconstruction of the coracoclavicular ligament with graft or synthetic materials.

This study showed better functional outcomes in patients with a Rockwood grade III ACJ injury managed with CC ligament repair or reconstruction than ACJ fixation with hardware. Pin migration, pain, and osteoarthritis were the most frequently occurring complications in group 1 and are in keeping with previous studies [44,45]. Despite level III evidence supporting good outcomes [12,31], the use of K-wires and pins has gone into disrepute, and both are now contraindicated due to the frequency of their complications [46,47]. The most prevalent complication reported with hook plate use was the requirement for further surgery to remove the plate, acromial osteolysis, and wound infections due to the open nature of this procedure. Our study showed that CC ligament repair with a loop or suspensory devices reported the lowest complication rates. Ligament ossification, dislocation of TightRope®, and Bosworth screw breakage were the frequently occurring complications in group 2 patients. Also, the varied operative outcomes observed in the management of Rockwood grade III injuries have been attributed to the heterogeneity of injury patterns seen in this group and the poor intra-observer and inter-observer reliability of the Rockwood classification system, especially in higher-grade injuries. Beitzel et al. have gone a step further to propose the addition of a grade IIIA and type IIIB (horizontally stable and unstable injuries) to a modified Rockwood classification to aid surgical decision-making [48].

Limitations

This review has substantial limitations. Although there were two level I studies, most of the included studies were retrospective and level III evidence. Furthermore, several of the studies had shown evident bias in selecting patients, while others did not include all complications. Some studies did not report the outcome measures being assessed. In addition, several studies did not consider or report radiographic outcomes. Finally, a few of the surgical techniques described have since become obsolete, while others such as the use of threaded Kirschner wires for intra-articular ACJ fixation are outrightly contraindicated today.

## Conclusions

The results of this study are in line with current biomechanical and clinical studies ﻿that show that the reconstruction of the CC ligament using autogenous or synthetic ligaments, such as the TightRope® and Surgilig/LARS^TM ^Ligament, as opposed to rigid fixation methods, is more effective for the management of acute ACJ dislocations.

Due to the lack of sufficient level I evidence available in the current literature, appropriately powered randomized control studies are required to compare the various CC ligament repair/reconstruction techniques to determine a gold standard for the surgical management of acute grade III ACJ dislocations.
